# β2-Adrenergic Receptor Signaling Pathway Stimulates the Migration and Invasion of Cancer Cells via Src Activation

**DOI:** 10.3390/molecules27185940

**Published:** 2022-09-13

**Authors:** Jae-Hoon Jeong, Hyun-Ji Park, Shin-Hyung Park, Yung-Hyun Choi, Gyoo-Yong Chi

**Affiliations:** 1 Department of Pathology, College of Korean Medicine, Dong-eui University, Busan 47227, Korea; 2 Department of Biochemistry, College of Korean Medicine, Dong-eui University, Busan 47227, Korea

**Keywords:** β2-adrenergic receptor, hepatocellular carcinoma, breast cancer, Src, migration, invasion

## Abstract

Chronic stress has been reported to stimulate the release of catecholamines, including norepinephrine (NE) and epinephrine (E), which promote cancer progression by activating the adrenergic receptor (AR). Although previous studies showed that β2-AR mediated chronic stress-induced tumor growth and metastasis, the underlying mechanism has not been fully explored. In this study, we aimed to investigate the molecular mechanism by which β2-AR exerts a pro-metastatic function in hepatocarcinoma (HCC) cells and breast cancer (BC) cells. Our results showed that Hep3B human HCC cells and MDA-MB-231 human BC cells exhibited the highest *ADRB2* expression among diverse HCC and BC cell lines. NE, E, and isoprenaline (ISO), adrenergic agonists commonly increased the migration and invasion of Hep3B cells and MDA-MB-231 cells. The phosphorylation level of Src was significantly increased by E/NE. Dasatinib, a Src kinase inhibitor, blocked E/NE-induced migration and invasion, indicating that AR agonists enhanced the mobility of cancer cells by activating Src. *ADRB2* knockdown attenuated E/NE-induced Src phosphorylation, as well as the metastatic ability of cancer cells, suggesting the essential role of β2-AR. Taken together, our results demonstrate that chronic stress-released catecholamines promoted the migration and invasion of HCC cells and BC cells via β2-AR-mediated Src activation.

## 1. Introduction

The relationship between chronic stress and cancer progression has been an interesting topic in cancer research. It is widely accepted that stressful life circumstances accelerate the initiation and progression of cancer [1]. The β-adrenergic receptor (AR) signaling pathway is one of the key mediators of this event. Under stressful conditions, catecholamines, including norepinephrine (NE) and epinephrine (E), are increased not only in the blood but also in tumor tissue, because catecholaminergic nerve fibers extensively innervate the tumor parenchyma [2,3]. The activation of β-AR by stress-released catecholamines stimulates downstream effectors such as protein kinase A (PKA) and exchange protein activated by adenylyl cyclase (EPAC). This, in turn, phosphorylates diverse transcription factors, including signal transducer and activator of transcription 3 (STAT3), NF-кB, and cAMP-responsive element binding protein (CREB), to regulate the expression of target genes involved in apoptosis/anoikis, inflammation, immune responses, and angiogenesis [2,3]. β-adrenergic activation also promotes cancer metastasis by stimulating cancer cell motility, invasion, and epithelial-mesenchymal transition (EMT) [2,3]. Consistently, β-AR antagonists (β-blockers) have been reported to exert antitumor activities in diverse types of cancers. For example, propranolol, a non-selective β-blocker, reduced chronic stress-induced pancreatic tumor burden and prolonged the survival of mice [4]. The long-term use of propranolol reduced the risk of hepatocellular carcinoma (HCC) by more than 50% and improved the overall survival of HCC patients [5,6].

Among the three subtypes of β-AR, which include β1, β2, and β3, β2-AR has been considered the most important target for cancer therapy. This idea was supported by the clinical observations that β1-selective blockers primarily used to treat cardiovascular diseases did not show antitumor effects as non-selective β-blockers [7,8]. β2-AR is highly expressed in various tumor tissues and is closely associated with poor patient prognosis [9,10]. The polymorphism of *ADRB2*, a gene encoding β2-AR, was reported to be a marker for predicting treatment responses and the prognosis of patients with cancer [11]. In mouse models, chronic restraint stress stimulated angiogenesis and promoted tumor growth in pancreatic cancer and ovarian cancer by activating β2-AR [12,13]. A β2-specific antagonist reversed restraint stress-induced invasion and lymph node metastasis in gastric cancer, suggesting that targeting β2-AR could be an attractive strategy to treat cancer [14].

The Src family kinases are nonreceptor tyrosine kinases implicated in tumorigenesis and cancer progression by regulating tumor growth, angiogenesis, and metastasis [15]. Src is activated by focal adhesion kinase (FAK) and a variety of receptor tyrosine kinases, including epidermal growth factor receptor (EGFR), HER2, and vascular endothelial growth factor receptor (VEGFR) [15]. Src can also be activated by β-AR via the PKA/β-AR kinase (BARK)/β-arrestin axis [3]. Armaiz-Pena et al. [16] reported that Src phosphorylation mediated NE-induced migration and invasion in ovarian cancer cells, suggesting a critical role of the β-AR/Src axis in promoting tumor metastasis. However, the involvement of β2-AR and Src in regulating the metastatic ability of hepatocellular carcinoma (HCC) cells and breast cancer (BC) cells has not been explored yet. In this study, we have investigated the underlying mechanism by which the β2-AR signaling pathway promotes the migration and invasion of HCC and BC cells, focusing on the role of Src.

## 2. Results

### 2.1. Expression of ADRB2 in Human HCC and BC Cell Lines

As we focused on the role of the β2-AR signaling pathway in the metastatic ability of HCC and BC cells, we first investigated the mRNA expression of *ADRB2* in various human HCC and BC cell lines. Among HCC cell lines with different genetic backgrounds, including Hep3B, Huh-7, PLC/PRF/5, SNU-475, and HepG2, the Hep3B cell line showed the strongest expression of *ADRB2* (Figure S1A in Supplementary Materials). Among the 5 BC cell lines, including MDA-MB-231, T47D, BT20, MCF7, and SK-BR-3, the triple-negative BC (TNBC) MDA-MB-231 cells expressed the highest level of *ADRB2* (Figure S1B). Similarly, it has been reported that the TNBC cell lines exhibited a higher expression of β2-AR than other cell lines [17]. Therefore, we chose Hep3B and MDA-MB-231 cell lines for further experiments.

### 2.2. Effect of β-AR Signaling Pathway on the Migration and Invasion of Human HCC and BC Cells

**Figure 1:**
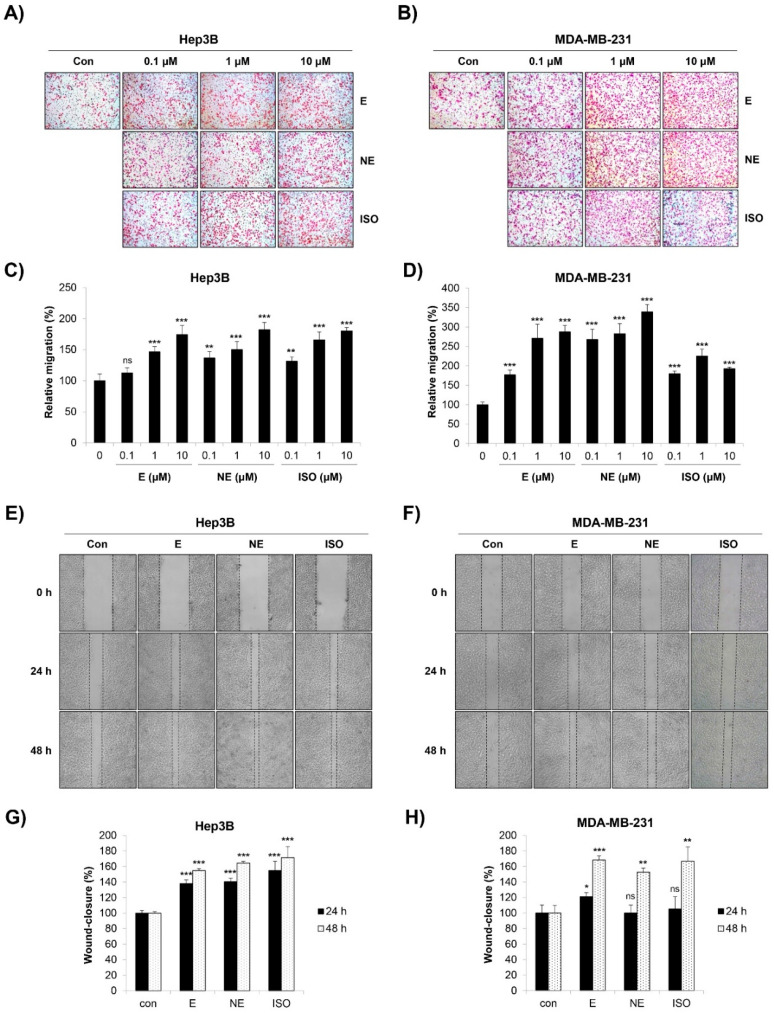
Effect of adrenergic agonists on the migration of Hep3B and MDA-MB-231 cells. (**A**–**D**) Hep3B human HCC cells (**A**,**C**) and MDA-MB-231 human BC cells (**B**,**D**) suspended in serum-free media were seeded onto the inserts of transwell plates and treated with either E or NE or ISO at 0.1–10 μM. Medium containing 10% FBS was filled in the bottom chamber as a chemoattractant. After 24 h of incubation, the migrated cells were stained and photographed (×100 magnification). (**A**,**B**) Representative images from triplicate analyses are shown. (**C**,**D**) Relative migration was calculated by counting the stained cells. (**E**–**H**) Confluent cell layers of Hep3B human HCC cells (**E**,**G**) and MDA-MB-231 human BC cells (**F**,**H**) were scratched for the wound-healing assay. The cells were then treated with either E (10 μM) or NE (1 μM) or ISO (10 μM) in serum-free medium. (**E**,**F**) Wound closure was photographed immediately, and at 24 h and at 48 h post-wound generation (×50 magnification). Representative images from triplicate analyses are shown. (**G**,**H**) The proportion of wound closed was calculated. The data are expressed as the mean ± SD of three independent experiments. Significance was determined by the Student’s *t*-test (ns, not significant; * *p* < 0.05; ** *p* < 0.01; *** *p* < 0.001 vs. untreated controls). HCC, hepatocellular carcinoma; BC, breast cancer; E, epinephrine; NE, norepinephrine; ISO, isoprenaline.

**Figure 2:**
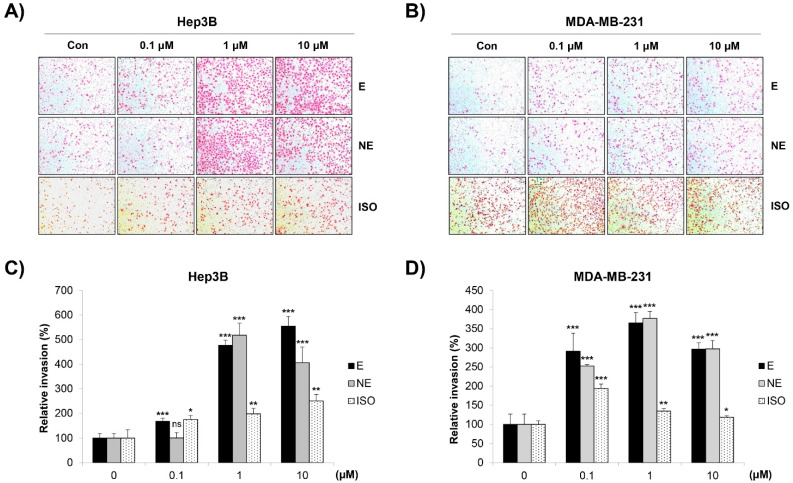
Effect of adrenergic agonists on the invasive capacity of Hep3B and MDA-MB-231 cells. (**A**–**D**) Hep3B human HCC cells (**A**,**C**) and MDA-MB-231 human BC cells (**B**,**D**) suspended in serum-free media were seeded onto the inserts of transwell plates and treated with either E or NE or ISO at 0.1–10 μM. The inner membrane of the insert was coated with Matrigel. Medium containing 10% FBS was added to the bottom chamber as a chemoattractant. After 24 h of incubation, the invaded cells were stained and photographed (×100 magnification). (**A**,**B**) Representative images from triplicate analyses are shown. (**C**,**D**) Relative invasion was calculated by counting the stained cells. The data are expressed as the mean ± SD of three independent experiments. Significance was determined by the Student’s *t*-test (ns, not significant; * *p* < 0.05; ** *p* < 0.01; *** *p* < 0.001 vs. untreated controls). HCC, hepatocellular carcinoma; BC, breast cancer; E, epinephrine; NE, norepinephrine.

We next investigated the influence of the β-AR signaling pathway on the migration of Hep3B and MDA-MB-231 cells. The transwell migration assay results showed that the number of migrated cells was significantly increased by epinephrine (E) and norepinephrine (NE), non-selective AR agonists. The β-AR agonist isoprenaline (ISO) also promoted the cell migration of Hep3B and MDA-MB-231 cells (Figure 1A–D). Similar results were obtained when the cell mobility was assessed by the wound-healing assay. The cells treated with E/NE/ISO closed the wound area faster than the control cells (Figure 1E,F). In addition, these adrenergic agonists greatly increased the invasive capacity of Hep3B and MDA-MB-231 cells (Figure 2A–D). To exclude the possibility that the increased migration and invasion were due to the proliferation-promoting effect of adrenergic agonists as reported previously [18], we investigated whether E/NE/ISO regulated the growth of HCC and BC cells. Our results clearly showed that E/NE/ISO had no or only marginal effects on the proliferation of Hep3B and MDA-MB-231 cells (Figure S2A,B). Since E and NE bind to both α-AR and β-AR, we next determined which type of receptor was responsible for E/NE-induced cancer cell migration. We found that propranolol, a non-selective β-blocker, and ICI-118,551, a β2-AR antagonist, completely impaired E/NE-induced migration in Hep3B and MDA-MB-231 cells, demonstrating that β2-AR was essential for mediating the migration-stimulating effect of E and NE (Figure S3A,B). Collectively, our observations suggest that E/NE/ISO promoted the migration and invasion of human HCC and BC cells via the β-AR signaling pathway, which is consistent with the results of previous studies [19,20,21].

### 2.3. Role of Src in E/NE-Induced Migration and Invasion in Human HCC and BC Cells

**Figure 3:**
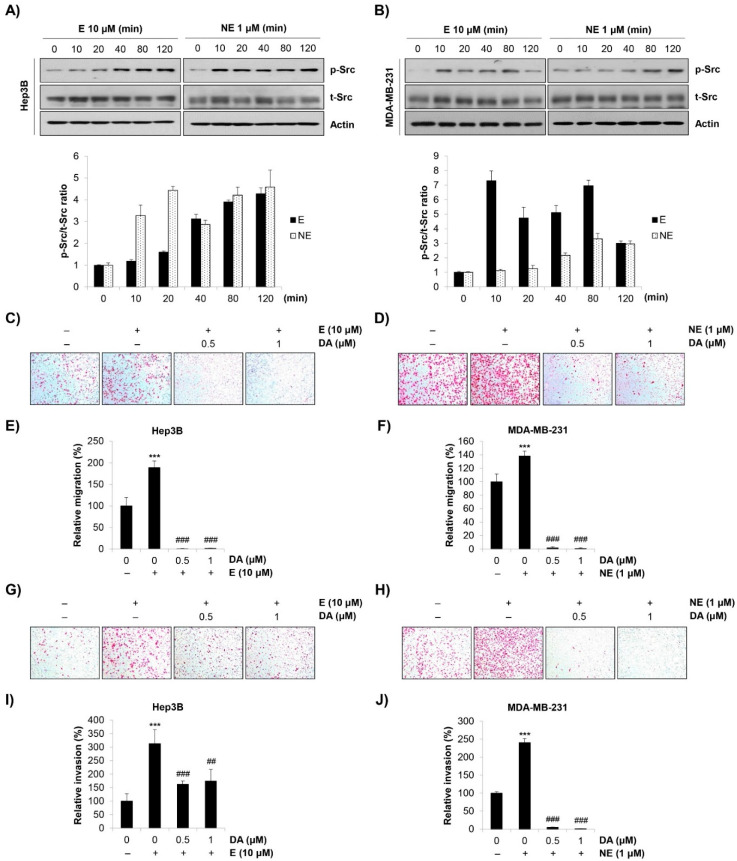
E/NE-induced Src activation stimulates the migration and invasion of Hep3B and MDA-MB-231 cells. (**A**,**B**) Hep3B human HCC cells (**A**) and MDA-MB-231 human BC cells (**B**) were treated with E (10 μM) or NE (1 μM) for the indicated times. Phosphorylated Src and total Src were detected by Western blot analysis. Actin was used as the internal control. The representative images of duplicate experiments are shown (upper). The ratio of phosphorylated/total Src was calculated using ImageJ software after normalization to actin (lower). (**C**–**J**) Hep3B cells (**C**,**E**,**G**,**I**) and MDA-MB-231 cells (**D**,**F**,**H**,**J**) were seeded in the inserts of transwell plates and co-treated with adrenergic agonists (10 μM E for Hep3B; 1 μM NE for MDA-MB-231) and dasatinib (0.5–1 μM). After 24 h of incubation, the migrated (**C**–**F**) or invaded (**G**–**J**) cells were stained and photographed (×100 magnification). (**C**,**D**,**G**,**H**) Representative images from triplicate analyses are shown. (**E**,**F**,**I**,**J**) Relative migration (**E**,**F**) and invasion (**I**,**J**) was calculated by counting the stained cells. The data are expressed as the mean ± SD of three independent experiments. Significance was determined by the Student’s *t*-test (*** *p* < 0.001 vs. untreated controls; ^##^ *p* < 0.01, ^###^ *p* < 0.001 vs. E/NE-treated cells). HCC, hepatocellular carcinoma; BC, breast cancer; E, epinephrine; NE, norepinephrine; DA, dasatinib.

Src plays a crucial role in regulating cell-cell adhesion and focal adhesion, thereby influencing cellular migration and invasion [15]. Since Src can be activated by the β-AR signaling pathway [3], we focused on the role of Src in the E/NE-induced metastatic ability of human HCC and BC cells. As shown in Figure 3A,B, Src was phosphorylated in response to E/NE stimulation. In addition, the E/NE-induced migration and invasion of Hep3B and MDA-MB-231 cells were completely abrogated by dasatinib, a Src kinase inhibitor (Figure 3C–J). The concentration range of dasatinib (0.5–1 μM) that showed anti-migration and anti-invasion effects in cancer cells only negligibly affected cell viability, which indicated a minimal influence of cytotoxicity on the anti-metastatic effects of dasatinib (Figure S4A,B). Taken together, our observations suggest that E/NE promoted the migration and invasion of human HCC and BC cells by activating Src.

### 2.4. β2-AR/Src Axis Mediated E/NE-Induced Migration and Invasion in HCC and BC Cells

**Figure 4:**
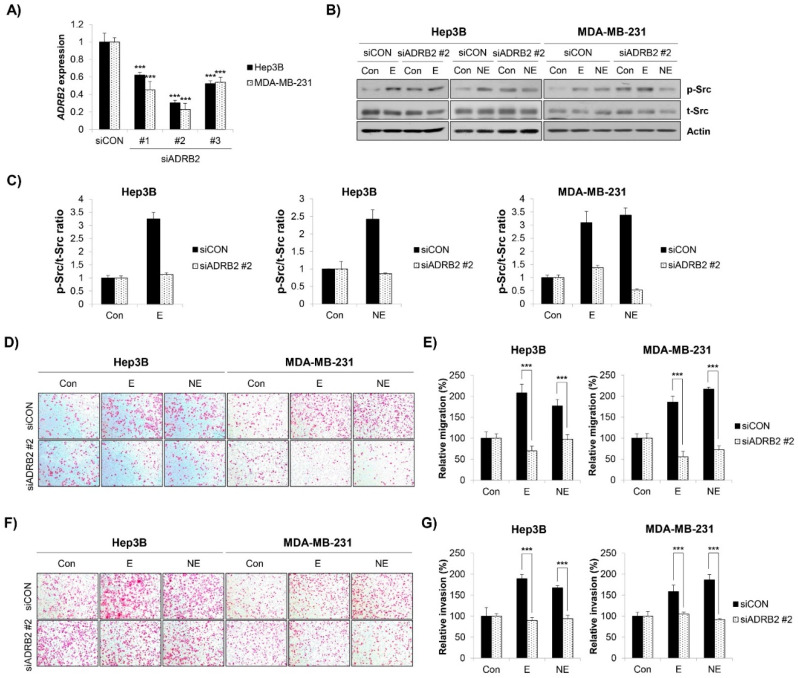
β2-AR is involved in E/NE-induced Src activation and the metastatic ability of Hep3B and MDA-MB-231 cells. (**A**–**G**) Hep3B human HCC cells and MDA-MB-231 human BC cells were transfected with control siRNA or *ADRB2* siRNA #1, #2, and #3 (see Materials and Methods). (**A**) At 24 h post-transfection, the expression of *ADRB2* was assessed by real-time PCR. (**B**,**C**) At 24 h post-transfection, the cells were treated with E (10 μM) or NE (1 μM) for 2 h. Phosphorylated Src and total Src were detected by Western blot analysis. Actin was used as the internal control. The representative images of duplicate experiments are shown (**B**). The ratio of phosphorylated/total Src was calculated using ImageJ software after normalization to actin (**C**). (**D**–**G**) At 24 h post-transfection, the cells were seeded again onto the inserts of transwell plates and treated with E (10 μM) or NE (1 μM). After 24 h of incubation, the migrated (**D**,**E**) or invaded (**F**,**G**) cells were stained and photographed (×100 magnification). (**D**,**F**) Representative images from triplicate analyses are shown. (**D**,**F**) Relative migration (**E**) and invasion (**G**) of E/NE-treated cells compared with untreated control cells was calculated by counting the stained cells. The data are expressed as the mean ± SD of three independent experiments. Significance was determined by the Student’s *t*-test (*** *p* < 0.001 vs. relative control). HCC, hepatocellular carcinoma; BC, breast cancer; E, epinephrine; NE, norepinephrine.

We next investigated the role of β2-AR on E/NE-induced Src activation. As β2-AR has been the most important target for cancer therapy among the three subtypes of β-AR, we knocked down *ADRB2* in Hep3B and MDA-MB-231 cells using *ADRB2* siRNA. Among the three kinds of *ADRB2* siRNA, siRNA #2 showed the strongest knockdown efficiency (Figure 4A). Transfection with *ADRB2* siRNA #2 completely blocked E/NE-induced Src phosphorylation in Hep3B and MDA-MB-231 cells, suggesting that β2-AR mediated Src activation (Figure 4B,C). Consistently, E/NE-induced migration and invasion in Hep3B and MDA-MB-231 cells were totally impaired by *ADRB2* knockdown (Figure 4D–G). Taken together, our observations suggest that β2-AR was essential for E/NE-induced Src phosphorylation as well as the subsequent migration and invasion of human HCC and BC cells.

## 3. Discussion

In the current study, we explored the underlying mechanism by which adrenergic agonists promoted migration and invasion in HCC cells and BC cells. We found that E/NE/ISO commonly increased the migratory and invasive abilities of HCC cells and BC cells. E/NE-induced Src activation was responsible for the increased migration and invasion and was blocked by *ADRB2* knockdown. To the best of our knowledge, this was the first study suggesting the crucial role of the β2-AR/Src axis in the metastatic ability of HCC cells and BC cells. Although previous studies implicated the activation of the β2-AR signaling pathway in chronic stress-related cancer progression, a specific role of Src in this event has not been fully understood. Armaiz-Pena et al. [16] reported that NE stimulated the growth and invasion of ovarian cancer by activating Src. However, the involvement of the β2-AR/Src axis in regulating the migration and invasion of HCC cells and BC cells has not been explored yet, highlighting the novelty of this study.

The reasons why we focused especially on HCC and BC are as follows. Regarding HCC, propranolol was reported to reduce the risk for HCC and improve the prognosis of HCC patients [5,6]. In a cell-based study, β-AR expression was higher in HCC cells than in normal liver cells, and propranolol down-regulated their expression and induced apoptosis in HCC cells [18]. In addition, NE-induced HCC invasion and anoikis inhibition was primarily mediated by β2-AR [20]. Regarding the BC, propranolol-treated patients (but not β1-selective blocker atenolol-treated patients) were less likely to present with advanced-stage tumors and showed better prognosis than non-users, suggesting that the blockage of β2-AR contributed to the anticancer effects of propranolol [8]. The importance of the β-AR signaling pathway has been emphasized especially in TNBC cells expressing higher levels of β2-AR than hormone receptor-positive BC cells [17]. β-blocker improved recurrence-free survival and reduced metastases in TNBC patients [17]. According to the theory of traditional Oriental medicine, chronic psychological stress primarily promotes the occurrence and progression of liver cancer and breast cancer by inducing qi stagnation. Qi stagnation is the key pathogenesis of a variety of diseases, including mental disorders, fibroids, and cancer, by producing blood stasis and phlegm [22]. These previous studies and theories support our hypothesis that chronic stress-activated β2-AR signaling plays a crucial role in promoting the migration and invasion of HCC cells and BC cells.

Despite the novelty of the current study, our findings have some limitations that should be considered. First, our results should be validated in more cell lines because we used just two cell lines for predicting the role of the β2-AR/Src axis in HCC cells and BC cells. Second, the involvement of other AR subtypes, including α-AR, β1-AR, and β3-AR, in regulating the migratory and invasive capacity of HCC cells and BC cells was not ascertained. NE/E-induced cell invasion was reportedly mediated by both α1-AR and β2-AR in HCC [20]. Armaiz-Pena et al. also reported that not only β2-AR but β1-AR was responsible for NE-induced Src phosphorylation [16]. Therefore, the role of other AR subtypes in modulating the behaviors of HCC and BC should be further explored in future studies.

Taken together, the current study demonstrated that the β2-AR/Src axis is critically involved in adrenergic agonist-induced migration and invasion in human HCC cells and BC cells. Our results suggest that the β2-AR/Src axis could be an attractive target to manage the chronic stress-induced progression of HCC and BC.

## 4. Materials and Methods

### 4.1. Reagents and Antibodies

Propranolol, ICI-118,551, E, and NE, purchased from Sigma-Aldrich (St Louis, MO, USA), were dissolved in distilled water (DW) at 100 mM. Isoprenaline (ISO) and dasatinib were also purchased from Sigma-Aldrich and reconstituted in dimethyl sulfoxide (DMSO; Amresco, Solon, OH, USA) at 100 mM and at 4 mM, respectively. The primary antibody against phospho-Src (Y416) was purchased from Cell Signaling Technology (Beverly, MA, USA). Primary antibodies against Src and actin were obtained from Santa Cruz Biotechnology (Santa Cruz, CA, USA). The anti-mouse and anti-rabbit secondary antibodies were purchased from Bethyl Laboratories (Montgomery, TX, USA) and Enzo Life Sciences (Farmingdale, NY, USA), respectively.

### 4.2. Cell Lines and Cell Culture

The Hep3B human HCC cell line and MDA-MB-231 human BC cell line were purchased from the American Type Culture Collection (ATCC; Rockville, MD, USA). MDA-MB-231 cells were grown in RPMI-1640 medium (WelGENE, Daegu, Korea) supplemented with 100 mL/L of fetal bovine serum (FBS; WelGENE), 100,000 U/L of penicillin (WelGENE), and 100 mg/L of streptomycin (WelGENE). Hep3B cells were cultured in Dulbecco’s Modified Eagle Medium (DMEM, WelGENE) supplemented as described above. The cells were cultured at 37 °C in a humidified atmosphere under 5% CO_2_.

### 4.3. Transwell Assay

For the transwell migration assay, Hep3B cells (2 × 10^4^ cells) or MDA-MB-231 cells (3 × 10^4^ cells) suspended in serum-free media were seeded onto the inserts of 24-well transwell plates (Corning Costar, Lowell, MA, USA), and treated with diverse adrenergic agonists with or without propranolol and dasatinib. The outer membrane of the inserts was coated with 0.1% gelatin (Sciencell, Carlsbad, CA, USA). Medium containing 10% FBS was added to the bottom chamber as a chemoattractant. After 24 h of incubation, the cells that migrated through the membrane were stained with hematoxylin (Sigma-Aldrich), and photographed under a microscope (Leica, Wetzlar, Germany) at ×100 magnification. The transwell invasion assay was performed as described above, except that the inner membrane of the inserts was additionally coated with 300 μg/mL of Matrigel (BD Bioscience, San Jose, CA, USA).

### 4.4. Wound-Healing Assay

Cells were seeded in 6-well plates at a density of 8 × 10^5^ cells/well. When the cells reached 100% confluency, a vertical wound was made by scratching the cell monolayer with a 200-μL pipette tip. After washing twice with phosphate-buffered saline (PBS) to remove the floating cells, a fresh serum-free medium containing E or NE, or ISO was added to the wells. Wound closure was monitored for 48 h under a microscope (Leica), and photographed immediately, and at 24 h and at 48 h post-wound generation (×50 magnification).

### 4.5. Knockdown of ADRB2

siRNA oligonucleotides for the negative control (forward, 5′-UUCUCCGAACGUGUCACGUTT-3′, reverse, 5′-ACGUGACACGUUCGGAGAATT-3′), *ADRB2* #1 (forward, 5′-CGCCCAUAUUCUUAUGAAATT-3′, reverse, 5′-UUUCAUAAGAAUAUGGGCGTT-3′), *ADRB2* #2 (forward, 5′-GCCAUUACUUCACCUUUCATT-3′, reverse, 5′-UGAAAGGUGAAGUAAUGGCTT-3′), and *ADRB2* #3 (forward, 5′-CUGCCCUUCUUCAUCGUUATT-3′, reverse, 5′-UAACGAUGAAGAAGGGCAGTT-3′) were purchased from Genepharma (Shanghai, China). Cells were seeded in 6-well plates at a density of 3 × 10^5^ cells/well. After overnight stabilization, the cells were transfected with 100 pmol of siRNA using Lipofectamine 2000 (Invitrogen, Thermo Fisher Scientific, Waltham, MA, USA) according to the protocol provided by the manufacturer. At 24 h post-transfection, the cells were harvested or treated with E/NE for further experiments.

### 4.6. Reverse Transcription-Quantitative Polymerase Chain Reaction (RT-qPCR)

Total RNA was extracted using TRIzol reagent (Invitrogen; Thermo Fisher Scientific). First-strand cDNA was synthesized using CellScript All-in-One 5X First Strand cDNA Synthesis Master Mix (CellSafe, Yongin, Korea) as described in the manufacturer’s protocol. cDNA templates diluted 50-fold in nuclease-free water were subjected to qPCR analysis using SYBR green (Enzynomics, Daejeon, Korea) and a CFX Connect Real-Time PCR Detection System (Bio-Rad Laboratories, Hercules, CA, USA). The primer sequences were as follows: human *ADRB2* forward (5′-TTGCTGGCACCCAATAGAAGC-3′) and reverse (5′-CAGACGCTCGAACTTGGCA-3′) and human *GAPDH* forward (5′-GTCTCCTCTGACTTCAACAGCG-3′) and reverse (5′-ACCACCCTGTTGCTGTAGCCAA-3′). The annealing temperature was 55 °C.

### 4.7. Western Blot Analysis

Western blot methodology was described in detail elsewhere [23]. Briefly, total protein was extracted using RIPA buffer (Thermo Fisher Scientific) containing a protease inhibitor cocktail (Thermo Scientific) and phosphatase inhibitors (1 mM Na_3_VO_4_ and 100 mM NaF). Protein (20 µg) was separated by sodium dodecyl sulfate-polyacrylamide gel electrophoresis (SDS-PAGE) and transferred to polyvinyl difluoride (PVDF) membranes (Millipore, Bedford, MA, USA). After blocking with 3% bovine serum albumin (BSA, GenDEPOT, Katy, TX, USA), the membrane was probed with primary antibodies (1:1000 dilution), followed by anti-rabbit or anti-mouse secondary antibodies (1:5000 dilution). Protein expression was detected by the D-Plus ECL Femto System (Donginbio, Seoul, Korea) according to the manufacturer’s instructions.

### 4.8. Statistical Analysis

The data are presented as the mean ± standard deviation (SD). Statistical comparisons between 2 groups were performed by a paired Student’s *t*-test. *p* < 0.05 was considered statistically significant.

## Data Availability

The data presented in this study are available on request from the corresponding author.

## Supplementary Materials

The following supporting information can be downloaded at: https://www.mdpi.com/article/10.3390/molecules27185940/s1, Figure S1: *ADRB2* expression in diverse human HCC cells and human BC cells; Figure S2: Effect of adrenergic agonists on the proliferation of Hep3B cells and MDA-MB-231 cells; Figure S3: Effect of propranolol on the E/NE-induced migration of Hep3B cells and MDA-MB-231 cells; Figure S4: Effect of dasatinib on the viability of Hep3B cells and MDA-MB-231 cells.
