# Supplementary material for: β2-Adrenergic Receptor Signaling Pathway Stimulates the Migration and Invasion of Cancer Cells via Src Activation
Source: Molecules. 2022 Sep 13;27(18):5940. doi: 10.3390/molecules27185940 (PMC9503488; doi:10.3390/molecules27185940)
Supplement: Supplementary file 1 [file molecules-27-05940-s001.zip › molecules-1904265-supplementary.pdf]

Supplementary Materials and Methods

Cell lines and cell culture

PLC/PRF/5, SNU-475, HepG2 human HCC cell lines, and BT20, MCF7 human BC cell lines were purchased from the American Type Culture Collection (ATCC; Rockville, MD, USA). The Huh-7 human HCC cell line was a kind gift from Professor Yun-Han Lee (Keimyung University, Korea). Both T47D and SK-BR-3 human BC cell lines were purchased from the Korean Cell Line Bank (Seoul, Korea). PLC/PRF/5, SNU-475, BT20, MCF7, T47D, and SK-BR-3 cells were cultured in RPMI-1640 medium (WelGENE, Daegu, Korea) supplemented with 100 mL/L of fetal bovine serum (FBS; WelGENE), 100,000 U/L of penicillin (WelGENE), and 100 mg/L of streptomycin (WelGENE). The other cell lines, including HepG2 and Huh-7 cells, were cultured in Dulbecco's Modified Eagle Medium (DMEM, WelGENE) supplemented as described above. The cells were cultured at 37 °C in a humidified atmosphere under 5% CO_2.

MTT assay

Cells were seeded onto 96-well plates at a density of 4×10^3 cells/well. The cells were stabilized overnight and treated with the indicated drugs (E, NE, ISO, and dasatinib) for 24 h, followed by the addition of 3-(4,5-dimethylthiazol-2-yl)-2,5-diphenyltetrazolium bromide (MTT; Duchefa, Haarlem, The Netherlands) solution to the culture medium at 0.4 mg/mL. After 2 h of incubation, the supernatant was discarded and the MTT formazan was dissolved in 100 μL of DMSO. When the formazan was thoroughly solubilized, the absorbance of each well was measured using a microplate reader (SpectraMax M3; Molecular Devices, San Jose, CA, USA) at a wavelength of 540 nm.

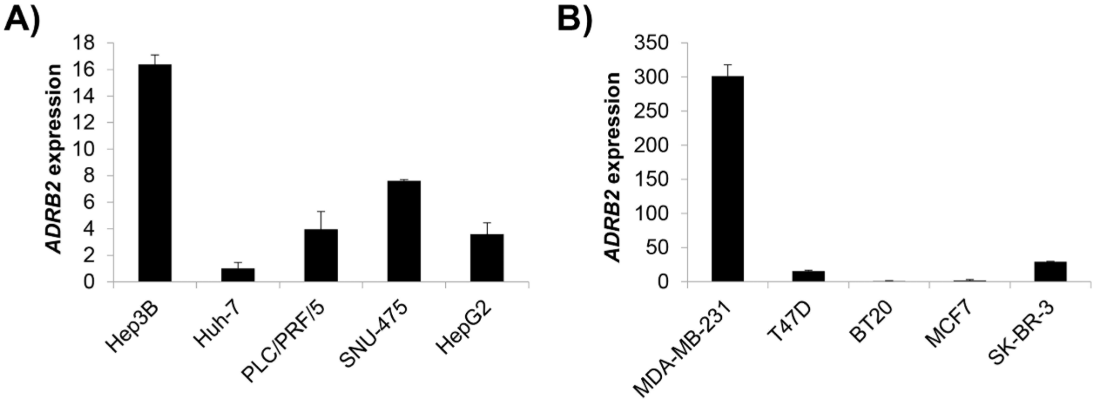

Supplementary Figure S1. ***ADRB2*** **expression in diverse human HCC cells and human BC cells.** The mRNA expression of *ADRB2* in human HCC cell lines and human BC cell lines was assessed by real-time PCR. HCC, hepatocellular carcinoma; BC, breast cancer.

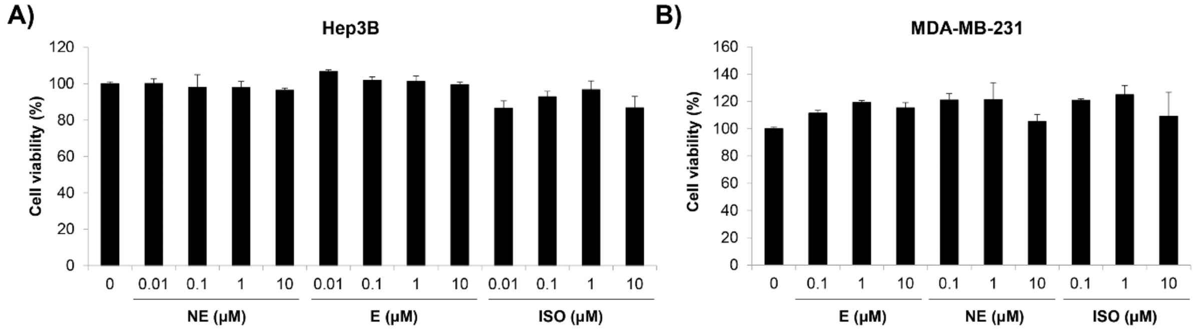

Supplementary Figure S2. Effect of adrenergic agonists on the proliferation of Hep3B cells and MDA-MB-231 cells. Hep3B human HCC cells (A) and MDA-MB-231 human BC cells (B) were treated with various concentrations of NE/E/ISO for 24 h. Cell viability was evaluated by the MTT assay. HCC, hepatocellular carcinoma; BC, breast cancer; E, epinephrine; NE, norepinephrine; ISO, isoprenaline.

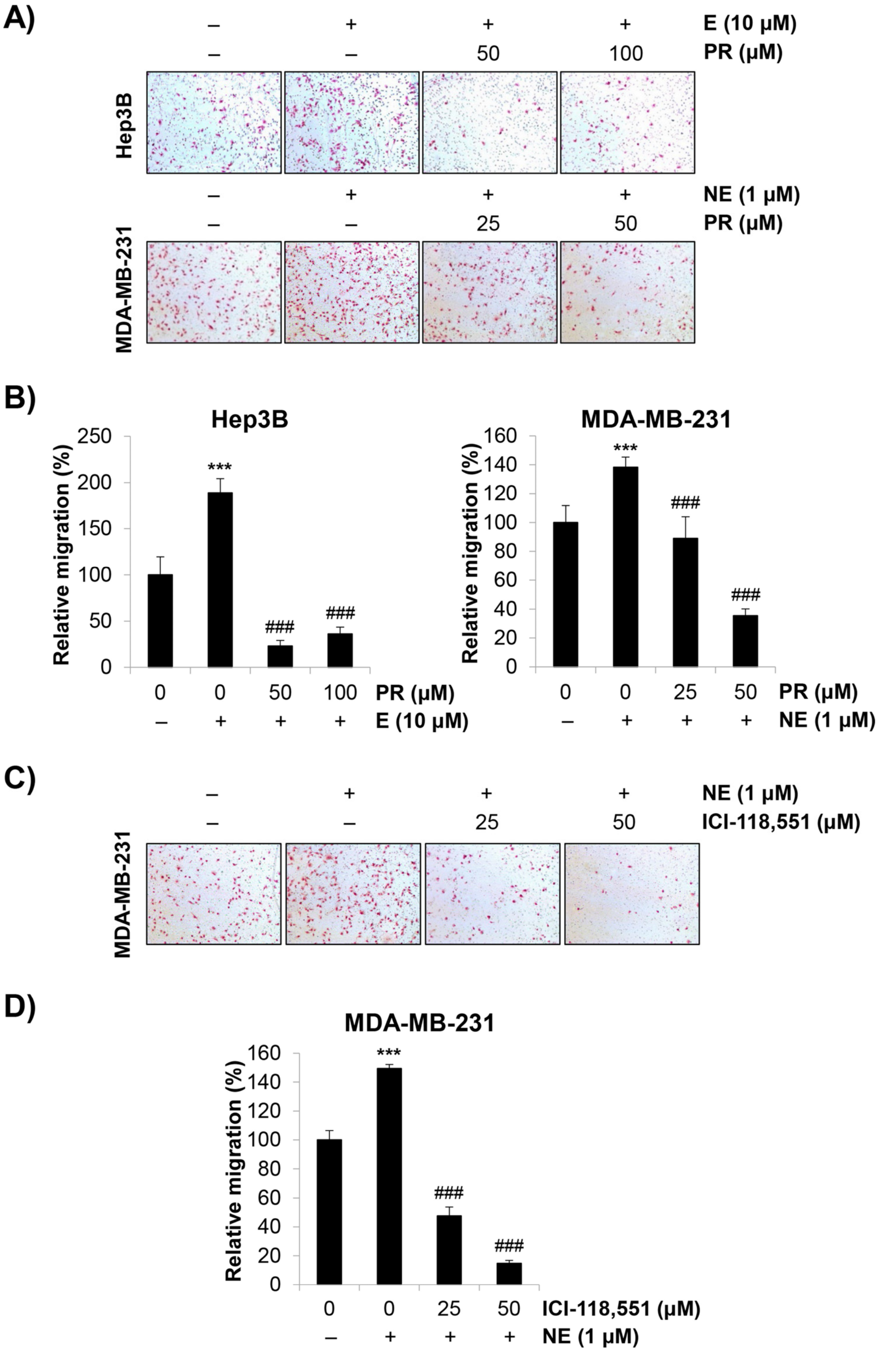

Supplementary Figure S3. Effect of propranolol on the E/NE-induced migration of Hep3B cells and MDA-MB-231 cells. (A and B) Hep3B human HCC cells and MDA-MB-231 human BC cells were seeded onto the inserts of transwell plates and co-treated with adrenergic agonists (10 μM E for Hep3B; 1 μM NE for MDA-MB-231)

and propranolol (50 – 100 μM). After 24 h of incubation, the migrated cells were stained and photographed (×100 magnification). (C and D) MDA-MB-231 cells were seeded onto the inserts of transwell plates and co-treated with NE (1 μM) and ICI-118,551 (25 – 50 μM). After 24 h of incubation, the migrated cells were stained and photographed (×100 magnification). (A and C) Representative images from triplicate analyses are shown. (B and D) Relative migration was calculated by counting the stained cells. The data are expressed as the mean ± SD of three independent experiments. Significance was determined by the Student's t-test ($^{***}P < 0.001$ vs. untreated controls, $^{###}P < 0.001$ vs. E/NE-treated cells). HCC, hepatocellular carcinoma; BC, breast cancer; E, epinephrine; NE, norepinephrine; PR, propranolol.

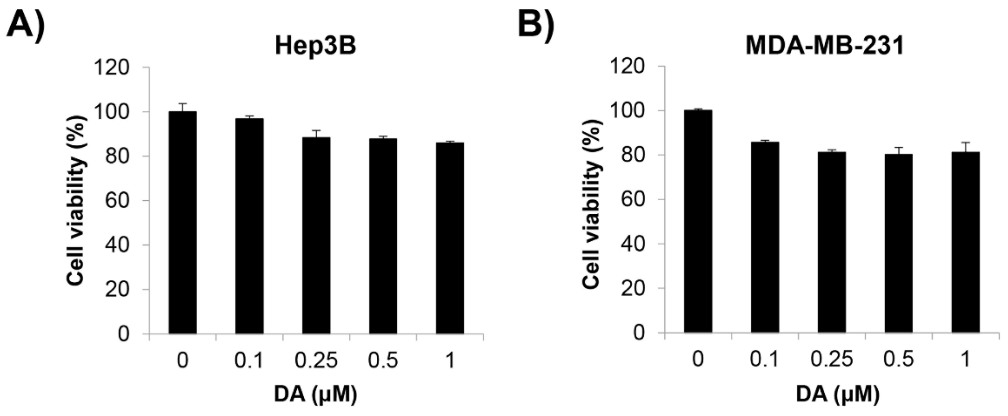

Supplementary Figure S4. Effect of dasatinib on the viability of Hep3B cells and MDA-MB-231 cells. Hep3B human HCC cells (A) and MDA-MB-231 human BC cells (B) were treated with various concentrations of dasatinib for 24 h. Cell viability was evaluated by the MTT assay. HCC, hepatocellular carcinoma; BC, breast cancer; DA, dasatinib.
